# Challenges in designing and running smouldering myeloma interventional clinical trials

**DOI:** 10.1002/jha2.880

**Published:** 2024-03-27

**Authors:** Catherine Lecat, Eileen M Boyle, Daniel Hughes, Lydia Lee, Dean Smith, Ceri Bygrave, Karthik Ramasamy, Kwee Yong

**Affiliations:** ^1^ Department of Haematology UCL Cancer Institute London UK; ^2^ Department of Clinical Haematology Nottingham University Hospitals NHS Trust Nottingham UK; ^3^ Department of Haematology University Hospital of Wales Cardiff UK; ^4^ Oxford Translational Myeloma Centre, NDORMS University of Oxford Oxford UK

**Keywords:** clinical trial, multiple myeloma, precursor disease, smouldering myeloma

1

Smouldering myeloma (SMM) is a heterogeneous, precursor condition to multiple myeloma (MM). SMM patients have either a serum M‐protein ≥30 g/L or clonal bone marrow (BM) plasma cells of 10–59% but the absence of myeloma‐defining events^1^. The current standard of care for SMM is observation. The risk of progression to MM is 10% / year for the first 5 years after diagnosis. However, those with high‐risk disease have > 50% chance of progression within 2 years based on the International Myeloma Working Group (IMWG) risk model [1]. Two clinical trials using lenalidomide showed a significant delay in MM progression and end‐organ damage [2, 3]. However, there are several challenges when designing interventional SMM trials (Figure 1).

**FIGURE 1:**
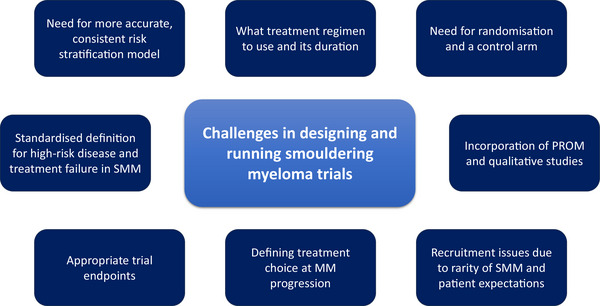
Challenges in designing and running smouldering myeloma (SMM) trials.

Firstly, there is a need for more accurate risk stratification to ascertain benefit over risk. Several SMM risk models have been developed, including the Mayo 20‐2‐20 model, IMWG model, PETHEMA model and PANGEA model [1, 4–6]. Whilst these models identify those at higher risk of progression, they were derived from retrospective data and are discordant [7]. SMM is a heterogenous, evolving condition and future models may require dynamic clinical, imaging or genomic biomarkers. Several genetic aberrations (e.g., NRAS, KRAS, MYC and APOBEC) have been identified to be associated with a higher risk of MM progression and could be used to improve current stratification models [8, 9]. To better understand this condition, the UK COSMOS trial (NCT05047107) is an ongoing multi‐centre, prospective, observational trial collecting clinical information, peripheral blood and BM samples from SMM patients longitudinally to determine the features of tumour genome and BM microenvironment, and to identify key drivers of MM progression.

Secondly, it is unclear what treatment regimen and duration is optimal for treating high‐risk SMM. Some trials utilise intensive therapy to eradicate MM clones, aiming for a cure. For example, the GEM‐CESAR trial uses carfilzomib, lenalidomide and dexamethasone as the induction regimen for autologous stem cell transplant, followed by consolidation and maintenance therapy. At 4 years post‐transplant, 23% of patients were negative for minimal residual disease (MRD) [10]. Other trials take a preventative approach by using immunotherapies, which may be more effective in an immune system that is less altered in SMM compared with MM. A study showed significant progression‐free survival (PFS) benefits using lenalidomide monotherapy compared to control despite a relatively low overall response rate (ORR) [3]. In the ongoing Immuno‐PRISM trial, all evaluable SMM patients receiving teclistamab, a bispecific T cell engager, achieved MRD negativity [11]. Other examples are shown in Table S1. Many of these trials lack a control arm, making it difficult to make meaningful comparisons or support regulatory approvals. Furthermore, some trials exclude patients with light chain‐only disease, and they often use different risk stratification criteria to define high‐risk, lacking a standardised approach to whom to treat. Current concepts of SMM trials generally include fixed‐duration treatment to minimise long‐term toxicities. Continuous treatment may cause a higher treatment discontinuation rate than fixed‐duration therapy and lead to refractory disease at progression.

Thirdly, the most appropriate endpoint for SMM studies remains undefined. Most trials use either ORR or PFS as the primary endpoint. Although response depth including MRD negativity is prognostic in MM, this may not hold true for SMM and further studies are required. The definition of treatment failure, which is generally defined by PFS in MM, remains debatable in SMM. Should this be biochemical progression or MRD resurgence? Or the presence of SLiM CRAB features (clinical progression) as most SMM trials have adopted? This definition would influence the time to offer second‐line treatment and as such, needs to be determined within the MM community internationally. Overall survival (OS) is arguably the most important endpoint for these patients, as early treatment may simply delay progression without prolonging life. DETER‐SMM (NCT03937635 – Daratumumab, lenalidomide and dexamethasone vs. lenalidomide and dexamethasone) is the only current phase III SMM trial that uses OS as the primary endpoint. However, using OS as a primary endpoint would require long follow‐up and may miss the benefits of delayed organ impairment or the need for intensive chemotherapy for MM. Moreover, it is important to explore other surrogate endpoints, especially those pertaining to morbidity (e.g., renal failure and bone disease).

Another consideration is treatment at progression. There is no consensus on what therapies to use when SMM trial patients progress, or whether they could be included in clinical trials for newly diagnosed MM. These patients could become refractory to multiple drug classes before even developing active myeloma. Studies focused on post‐frontline SMM treatment are needed. That being said, the standard of care for MM is constantly evolving, complicating this further.

Assessing quality‐of‐life (QoL) using patient‐reported outcome measures (PROM) is another crucial SMM trial endpoint. Maintaining QoL should be a treatment goal for these patients who are being treated for an asymptomatic condition. Potential treatment toxicities including infections, and additional hospital visits may significantly impact QoL. Patients may see these as disadvantages for joining the trial, causing recruitment issues. A lack of published SMM PROM studies meant that little is known about patients’ QoL or anxiety with a precursor condition, and their preferences towards its management. A study revealed that SMM patients had similar psychological distress as those with MM [12]. Finally, recruitment may be challenging due to the rarity of this condition. An Icelandic nationwide screening study iStopMM found an SMM prevalence of 0.53% in those ≥40 years old [13]. Only a proportion of these patients would have high‐risk disease (e.g. 9% of patients had high‐risk in the IMWG risk model study) and would agree to participate in a drug trial. It is also important to capture the reasons why patients decline trial participation, as well as their reasons for consent withdrawal, to better understand their attitudes towards treatment.

To conclude, a number of issues around SMM trials must be resolved to investigate treatment for this precursor condition. These include defining who to treat, how to treat, treatment goals and how to maintain QoL for patients on therapy. As the number of SMM trials increases globally, it is likely that the United Kingdom will soon open its first one, hopefully taking into account these challenges in its design. Finally, patient and public involvement is vital in shaping a successful trial to ensure adequate recruitment and optimal patient engagement throughout the trial.

## AUTHOR CONTRIBUTIONS

Catherine Lecat wrote the paper, and all authors revised and approved the final versions.

## CONFLICT OF INTEREST STATEMENT

The authors declare no conflict of interest.

## ETHICS STATEMENT

The authors have confirmed ethical approval statement is not needed for this submission.

## PATIENT CONSENT STATEMENT

The authors have confirmed patient consent statement is not needed for this submission.

## CLINICAL TRIAL REGISTRATION

The authors have confirmed clinical trial registration is not needed for this submission.

## Supporting information

Supporting Information

## Data Availability

N/A
