# Supplementary material for: Challenges in designing and running smouldering myeloma interventional clinical trials
Source: EJHaem. 2024 Mar 27;5(2):418–20. doi: 10.1002/jha2.880 (PMC11020121; doi:10.1002/jha2.880)
Supplement: Supplementary file 1 — Supporting Information [file JHA2-5-418-s001.docx]

**Supplementary table 1 - Smouldering myeloma clinical trials: completed or ongoing trials with interim reports**

| **Trial name** | **Phase** | **Patient no.** | **Recruitment period (months)** | **Risk stratification** | **Intervention** | **Primary endpoint** | **Response rates** | **PFS/OS or TTP** |
| --- | --- | --- | --- | --- | --- | --- | --- | --- |
| QuiRedex^1^ | III | 119 | Nov 2007 – Jun 2010 (32) | Paraprotein IgG ≥30g/L, IgA ≥20g/L + BMPC ≥10% or one factor with aPC ≥95% and immunoparesis | Lenalidomide + dexamethasone (Rd) vs observation | Time from randomisation to MM progression | Induction ORR 79%, ≥CR 14%  Maintenance ORR 90%, ≥CR 26% | Median TTP 9.0yr vs 2.1yr  Median OS NR vs 8.5yr |
| ECOG (NCT01169337)^2^ | III | 182 | Feb 2013 – Jul 2017 (54) | BMPC >10% or sheets of PCs and SFLC ratio <0.125 or >8 | R vs observation | Time from randomisation to MM progression | ORR 79%, ≥CR 0% in R arm | 3yr PFS 66% vs 91% |
| CENTAURUS^3^ | II | 123 | May 2015 – Jan 2017 (21) | BMPC ≥10%-<60% and one of paraprotein IgG ≥30g/L, IgA ≥20g/L, or urine M-protein >500mg/24h, or SFLC <0.126 or >8 and paraprotein 10-30g/L or iFLC ≥100mg/L | Daratumumab (Dara) monotherapy (short, intermediate and intense dosing schedules) | Rate of ≥CR 6 months after randomisation  PD/death rate per patient-year | ORR 38% (≥CR 0%), 56% (≥CR 12%) and 56% (≥CR 5%) in short, intermediate and intense arms respectively | Median OS NR in all arms  84-month OS 88%, 90%, 81% in short, intermediate and intense arms respectively |
| NCT01402284^4^ | Pilot | 12 | Jul 2011 – Oct 2013 (28) | PETHEMA criteria | Carfilzomib, lenalidomide + dexamethasone with lenalidomide maintenance (KRd-R) | ≥VGPR | ≥VGPR 100% (sCR 55%) | Estimated 4yr PFS 71% and OS 100% |
| NCT01572480^5^ | II | 54 | May 2012 – Jul 2020 (99) | PETHEMA, Mayo (2008) or Rajkumar et al (2015) criteria | KRd-R | MRD-ve CR rate | ORR 100%, ≥CR 75.9%, MRD-ve CR 70.4% | Estimated 8y PFS 91.2%  8y OS 100% |
| ASCENT^6^ | II | 87 | Jul 2018 – Nov 2021 (41) | Mayo (2018) or IMWG score | Dara-KRd (Induction, consolidation and maintenance) | sCR at the end of maintenance | Best ORR 97%, ≥CR 63%, sCR 37%, 84% MRD-ve | 3yr PFS 89.9% |
| GEM-CESAR^7^ | II | 90 | Jun 2015 – Jun 2017 (24) | Both BMPC ≥10% and paraprotein ≥30g/L or one of these and PETHEMA high-risk | KRd + melphalan ASCT + KRd + maintenance Rd | MRD-ve rate after ASCT, and at 3 and 5 years after ASCT | MRD-ve 63% after ASCT, 26% 4 years after ASCT | 5yr PFS 94% and OS 95% |
| NCT01484275^8^ | II | 85 | Mar 2012 – May 2015 (39) | BMPC ≥10%-<60% and one of paraprotein IgG ≥30g/L, IgA ≥20g/L, or urine M-protein >500mg/24h, or SFLC <0.126 or >8 and paraprotein 10-30g/L or iFLC ≥100mg/L | Siltuximab vs placebo | 1yr PFS | - | 1yr PFS 84.5% vs 74.4%  Median TTP NR vs 23.5 months |
| NCT01441973^9^ | II | 31 | Feb 2012 – Sep 2013 (20) | BMPC ≥10%-<60% and one of paraprotein IgG ≥30g/L, IgA ≥20g/L, or urine M-protein >500mg/24h, or SFLC <0.126 or >8 and paraprotein 10-30g/L or iFLC ≥100mg/L | Elotuzumab | Relationship between baseline proportion of BM-derived CD56^dim^ NK cells and maximal M protein reduction | ORR 10% | 2yr PFS 69% |
| NCT02697383^10^ | Pilot | 14 | Jun 2016 – Mar 2018 (21) | PETHEMA or Mayo (2008) criteria | Ixazomib + dexamethasone (Id) | ORR after 12 cycles | ORR 57% | 35% progression at 35 months |
| NCT02916771^11^ | II | 61 | Feb 2017 - Feb 2020 (37) | Rajkumar et al (2015) criteria | IRd with I or R maintenance | 2yr PFS | Post-induction ORR 92.3%, sCR 23.1% | Ongoing |
| ISAMAR^12^ | II | 61 | Feb 2017 – Oct 2022 (69) | Both PETHEMA high risk criteria | Isatuximab (Isa) ± R | ORR after 6 months of Isa | ORR 89% for IsaR | PFS 49 months, OS NR for Isa only  PFS/OS NR for IsaR |
| ITHACA^13^ | III | 23 | Recruiting | Mayo (2018) or PETHEMA criteria | IsaRd | Safety assessment and recommended dose in safety run-in, PFS in phase III | ORR 100%, ≥CR 30.4%, sCR 13% | Ongoing |
| NCT02603887^14^ | Pilot | 13 | Aug 2016 – Aug 2017 (12) | Mayo (2008), PETHEMA, or UAMS criteria | Pembrolizumab | ORR after 8 cycles | ORR 7.6%, sustained MRD-ve CR in the one responder | - |
| NCT01718899^15^ | I/IIa | 20 | - | Mayo (2008) | PVX-410 vaccine ± lenalidomide | Safety | Immunogenicity in 95% monotherapy and 100% in combination | - |
| Immuno-PRISM^16^ | II | 19 | Recruiting | Mayo (2008), IMWG risk score ≥9, PETHEMA, evolving-type SMM, high risk FISH abnormalities | Teclistamab vs Rd | CR rate | ORR 100% (CR in 42%) in teclistamab vs 66% in Rd arm | Ongoing |

**References for supplementary table 1**

1. Mateos MV, Hernández MT, Salvador C, et al. Lenalidomide-dexamethasone versus observation in high-risk smoldering myeloma after 12 years of median follow-up time: A randomized, open-label study. *Eur J Cancer*. 2022;174:243-250. doi:10.1016/j.ejca.2022.07.030

2. Lonial S, Jacobus S, Fonseca R, et al. Randomized Trial of Lenalidomide Versus Observation in Smoldering Multiple Myeloma. *Journal of Clinical Oncology*. 2020;38(11):1126-1137. doi:10.1200/JCO.19.01740

3. Landgren O, Chari A, Cohen YC, et al. Efficacy and Safety of Daratumumab (DARA) Monotherapy in Patients with Intermediate-Risk or High-Risk Smoldering Multiple Myeloma (SMM): Final Analysis of the Phase 2 Centaurus Study. *Blood*. 2023;142(Supplement 1):210-210. doi:10.1182/blood-2023-181765

4. Korde N, Roschewski M, Zingone A, et al. Treatment With Carfilzomib-Lenalidomide-Dexamethasone With Lenalidomide Extension in Patients With Smoldering or Newly Diagnosed Multiple Myeloma. *JAMA Oncol*. 2015;1(6):746. doi:10.1001/jamaoncol.2015.2010

5. Kazandjian D, Hill E, Dew A, et al. Carfilzomib, Lenalidomide, and Dexamethasone Followed by Lenalidomide Maintenance for Prevention of Symptomatic Multiple Myeloma in Patients With High-risk Smoldering Myeloma. *JAMA Oncol*. 2021;7(11):1678. doi:10.1001/jamaoncol.2021.3971

6. Kumar SK, Alsina M, Laplant B, et al. Fixed Duration Therapy with Daratumumab, Carfilzomib, Lenalidomide and Dexamethasone for High Risk Smoldering Multiple Myeloma-Results of the Ascent Trial. *Blood*. 2022;140(Supplement 1):1830-1832. doi:10.1182/blood-2022-168930

7. Mateos MV, Martínez-López J, Rodríguez-Otero P, et al. Curative Strategy (GEM-CESAR) for High-Risk Smoldering Myeloma (SMM): Post-Hoc Analysis of Sustained Undetectable Measurable Residual Disease (MRD). *Blood*. 2022;140(Supplement 1):292-294. doi:10.1182/blood-2022-159606

8. Brighton TA, Khot A, Harrison SJ, et al. Randomized, Double-Blind, Placebo-Controlled, Multicenter Study of Siltuximab in High-Risk Smoldering Multiple Myeloma. *Clinical Cancer Research*. 2019;25(13):3772-3775. doi:10.1158/1078-0432.CCR-18-3470

9. Jagannath S, Laubach J, Wong E, et al. Elotuzumab monotherapy in patients with smouldering multiple myeloma: a phase 2 study. *Br J Haematol*. 2018;182(4):495-503. doi:10.1111/bjh.15384

10. Mailankody S, Salcedo M, Tavitian E, et al. Ixazomib and dexamethasone in high risk smoldering multiple myeloma: a clinical and correlative pilot study. *Leuk Lymphoma*. 2022;63(11):2760-2761. doi:10.1080/10428194.2022.2095626

11. Nadeem O, Redd RA, Prescott J, et al. A Phase II Trial of the Combination of Ixazomib, Lenalidomide, and Dexamethasone in High-Risk Smoldering Multiple Myeloma. *Blood*. 2021;138(Supplement 1):2749-2749. doi:10.1182/blood-2021-149787

12. Manasanch EE, Korde N, Lee HC, et al. ISAMAR: Multicenter phase II single arm trial of isatuximab (ISA) with/without lenalidomide (LEN) in pts with high risk smoldering multiple myeloma (HRSMM). *Journal of Clinical Oncology*. 2023;41(16_suppl):8046-8046. doi:10.1200/JCO.2023.41.16_suppl.8046

13. Mateos MV, Rodriguez Otero P, Koh Y, et al. Isatuximab in Combination with Lenalidomide and Dexamethasone in Patients with High-Risk Smoldering Multiple Myeloma: Updated Safety Run-in Results from the Randomized Phase 3 Ithaca Study. *Blood*. 2022;140(Supplement 1):7317-7319. doi:10.1182/blood-2022-157302

14. Manasanch EE, Han G, Mathur R, et al. A pilot study of pembrolizumab in smoldering myeloma: report of the clinical, immune, and genomic analysis. *Blood Adv*. 2019;3(15):2400-2408. doi:10.1182/bloodadvances.2019000300

15. Nooka AK, Wang M (Luhua), Yee AJ, et al. Assessment of Safety and Immunogenicity of PVX-410 Vaccine With or Without Lenalidomide in Patients With Smoldering Multiple Myeloma. *JAMA Oncol*. 2018;4(12):e183267. doi:10.1001/jamaoncol.2018.3267

16. Nadeem O, Magidson S, Midha S, et al. Immuno-PRISM: A Randomized Phase II Platform Study of Bispecific Antibodies in High-Risk Smoldering Myeloma. *Blood*. 2023;142(Supplement 1):206-206. doi:10.1182/blood-2023-177954
